# Efficacy of Endodontic Files in Root Canal Retreatment: A Systematic Review of In Vitro Studies

**DOI:** 10.3390/jfb16080293

**Published:** 2025-08-14

**Authors:** Anna Soler-Doria, José Luis Sanz, Marcello Maddalone, Leopoldo Forner

**Affiliations:** 1 Department of Stomatology, Faculty of Medicine and Dentistry, Universitat de València, Gascó Oliag 1, 46010 Valencia, Spain; 2 Departamento di Medicina e Chirurgia, Università degli Studi di Milano-Bicocca, 20854 Milan, Italy

**Keywords:** root canal retreatment, endodontic files, systematic review

## Abstract

The success rate of root canal treatment is high, but it can fail. In these cases, orthograde root canal retreatment is often the treatment of choice, for which numerous biomaterials are available on the market, including endodontic files. This systematic review aimed to study the endodontic files available on the market and establish their efficacy in root canal retreatment. An electronic search of six different databases was performed, and in vitro experimental studies that studied root canal cleaning, debris extrusion, retreatment time, or dentinal damage produced with any of the comparison methods were selected. The quality of the studies was assessed with the QUIN scale. In total, 78 studies were included for the analysis, of which 9 had a high risk of bias, 53 had a moderate risk, and 16 had a low risk. The methods used to evaluate the efficacy of endodontic files in root canal retreatment are heterogeneous. Manual files produce more apical extrusion than rotary files. PTUR files are the most studied endodontic files. It is the file system that leaves the least residual filling material in the canal, takes the least time during retreatment, and removes the greatest amount of dentine. However, no file system achieved the complete removal of the root canal filling material. No consistent pattern emerged across studies when comparing rotary files with continuous and reciprocating rotation in terms of the removal of the filling material, the time required for de-obturation, dentine damage produced, or apical extrusion.

## 1. Introduction

Root canal treatment has a high success rate, ranging from 86 to 98% [1]. Nonetheless, it can sometimes fail. The main reason for endodontic failure is the persistence of bacterial populations, including Enterococcus faecalis [2], within the root canal system [3,4], followed by insufficient cleaning and the persistence of pulp tissue, inadequate root canal filling, coronal microfiltration, or the presence of untreated canals [1,4].

Upon root canal treatment failure, orthograde root canal retreatment [5] often appears as the treatment of choice [1,2,6,7], followed by endodontic microsurgery [1,4,7]. Successful retreatment is achieved by removing all previous root filling material and restoring apical patency in order to clean and shape the root canal again [8]. Mechanical, thermal, chemical, and even a combination of all three methods can be used to perform root canal retreatment, which is usually carried out with ultrasonic instruments [1] and hand or rotary files combined with the occasional use of heat or solvents [1,2]. The most commonly used solvents are chloroform, xylene, and orange oil [1]. Specific Ni-Ti files have been proposed [3] with the aim of improving shaping and cleaning efficacy and avoiding the apical extrusion of debris [9].

The aim of this systematic review was to perform a qualitative synthesis of in vitro studies that assess the efficacy of endodontic files for root canal retreatment.

## 2. Materials and Methods

This systematic review was conducted following the PRISMA 2020 guidelines, i.e., the Preferred Reporting Items for Systematic Reviews and Meta-Analysis [10]. The protocol for this systematic review was registered in the Open Science Framework (OSF) Registries and can be accessed using the following link: https://doi.org/10.17605/OSF.IO/XW58Z (accessed on 1 July 2025).

### 2.1. Study Question and Eligibility Criteria

The following PICOS question was established [10]: Are retreatment-specific files effective for root canal retreatment? The parameters were as follows: P, endodontic files; I, root canal retreatment; C, comparison between different files; O, parameters used to evaluate the efficacy of the tested files in root canal retreatment; S, in vitro studies.

Regarding the eligibility criteria, only experimental in vitro studies on endodontic files were eligible for inclusion. Additionally, studies were only eligible if they assessed one or more of the following parameters: the removal of filling material from inside the root canal, apical extrusion, dentine damage (the appearance of fissures or fractures, reduction in dentine thickness, etc.), intraoperative accidents, and working time. All other study types, studied materials, and assessed parameters were excluded.

### 2.2. Search Strategy and Study Selection

The advanced electronic search was conducted in January 2025 and was last updated in July 2025 in six databases: Medline, Embase, Scopus, Web of Science, SciELO, and Cochrane. The search strategy was based on the most previously cited descriptors in the field, as follows: ‘retreatment files’ AND (cleaning OR shaping OR extrusion OR debris OR dentin). The complete search strategy and findings per database are depicted in Table 1. No filters were used to limit the search results. No database-specific adaptations were needed.

The search results were exported to Mendeley (Elsevier, Amsterdam, The Netherlands) reference management software version 1.19.8 to discard duplicate records. Those that did not meet the eligibility criteria were discarded based on their titles and abstracts. After the first screening, the full manuscripts were screened to determine their inclusion in the study. Two reviewers (A.S-D. and L.F.) independently screened the titles and abstracts for eligibility and, subsequently, assessed the full texts of the potentially relevant reports. To quantify the consistency of their decisions, the Cohen’s kappa (κ) coefficient was calculated using SPSS version 26 (IBM Corp., Armonk, NY, USA). The kappa value was 0.89 (almost perfect agreement). Any discrepancies were resolved by discussion until a consensus was reached.

### 2.3. Data Extraction and Qualitative Synthesis

The data extraction included the following variables: authorship and year of publication, sample size and characteristics, preparation of each sample, studied files, use of solvent, and studied parameters. The extracted data were recorded in table format. A qualitative synthesis of the obtained results was subsequently performed, and this is presented in the Discussion Section.

### 2.4. Quality Assessment

To assess the quality and risk of bias of the selected articles, the QUIN rating scale, developed by Sheth et al. (2022) [11], was used. This scale consists of 12 items: clearly specified objectives, a detailed explanation of sample size calculation, a detailed explanation of sampling technique, the details of comparison group, a detailed explanation of methodology, the details of operators, the randomization, the methods of outcome measures, the details of outcome assessor, the blinding procedure, the statistical analysis and the presentation of results. Two reviewers (A.S-D. and L.F.) independently assessed the full texts of potentially relevant reports. Again, the Cohen’s kappa (κ) coefficient was calculated. The kappa value was 0.92 for quality assessment, reflecting almost perfect agreement between reviewers. Any discrepancies were also resolved by discussion until a consensus was reached.

## 3. Results

### 3.1. Results of the Search and Selection of Studies

**Figure 1:**
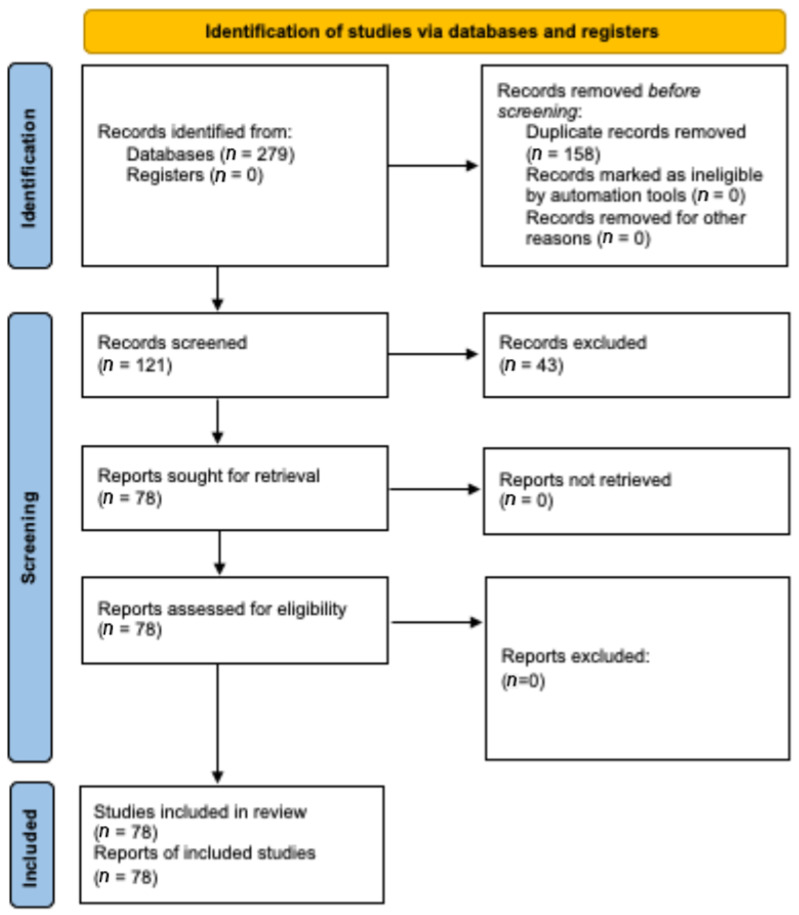
Study selection flow diagram. Based on the PRISMA 2020 flow diagram [10].

The final search yielded a total of 279 articles. A total of 158 duplicate records were discarded manually using the “search for duplicates” tool in Mendeley reference management software version 1.19.8. After the first screening of the remaining 121 records, a total of 43 records were excluded upon reading their titles and abstracts due to non-fulfilment of the eligibility criteria. The full texts of the resulting 78 records were retrieved and screened. All of them were considered eligible for the qualitative synthesis [1,2,3,4,5,6,7,8,9,12,13,14,15,16,17,18,19,20,21,22,23,24,25,26,27,28,29,30,31,32,33,34,35,36,37,38,39,40,41,42,43,44,45,46,47,48,49,50,51,52,53,54,55,56,57,58,59,60,61,62,63,64,65,66,67,68,69,70,71,72,73,74,75,76,77,78,79,80] (Figure 1).

### 3.2. Study Characteristics

The summary of the study characteristics and risk of bias are depicted in Table 2.

Table 3 shows the different files studied in the articles, the acronym used to refer to them throughout this article and the type of movement they perform. The most studied file is the PTUR (59 articles), followed by H-files (34), MTwoR, (26), and others.

### 3.3. Quality Assessment (Risk of Bias)

The quality analysis of the included studies was performed with the QUIN Quality Assessment Tool For In Vitro Studies [11]; it was concluded that 9 of the articles exhibited a high risk of bias, 53 exhibited a moderate risk, and 16 exhibited a low risk (Table 2). The full quality assessment, i.e., the fulfilment or non-fulfillment of each of the items in the QUIN Quality Assessment Tool For In Vitro Studies, is presented in Table A1.

## 4. Discussion

All the studies included in the present systematic review analyze the performance of a wide variety of files in vitro, whether they are intended for root canal retreatment or not, but are used for this purpose. The aim of this systematic review was to compare them and to qualitatively assess their efficacy in root canal retreatment. The decision to only include in vitro studies in the present work is based on the preliminary and novel nature of this review which, to our knowledge, is the first one to encompass and synthesize the available evidence regarding in vitro studies on endodontic files in root canal retreatment. In vitro studies are able to assess the tested variables in controlled conditions, limiting potential confounding variables, and serve as a base for future studies. However, this limits the extrapolation of their results into the clinical setting. Thus, this review may serve as a basis for researchers and clinicians to explore this specific topic.

### 4.1. Comparison of Hand Files vs. Rotary Files

The comparison of hand files with rotary files is a fairly common technique, as the long clinical history and long-term follow-up of hand files has meant that they are often used as an element of comparison. Thus, we see that numerous studies have compared the effects of manual files in retreatment with the use of rotary files [1,2,4,8,12,15,16,18,21,24,25,26,30,31,34,35,36,38,39,41,42,44,47,50,55,58,60,61,63,66,73].

The available evidence tends to report that rotary files remove more filling material than hand files in straight canals [1,2,21,24,26,34,36,47,50,61,66,73]. However, for Hasija et al. [52], the results from using H-files were better than those from using MtwoR and PTUR without solvent, although no significant differences were observed. These differences may be due to the evaluation method, because it was the only article that used a gray-scale-based evaluation. Singh et al. [78] observed that NeoEndoR files removed significantly more filling material compared to hand files, but there were no significant differences when comparing hand files with R-Endo files. However, in curved canals, the situation is different, as less residual filling material is left inside the canal with hand files than with rotary files [12].

When teeth were studied in thirds, it was observed that, in the apical third, where more residual material tends to remain [18], H-files work better [24,38].

Interestingly, with regards to apically extruded GP, the situation is different and depends more on the file used and less on whether it is manual or rotary. In this manner, H and MtwoR files have been found to remove apically overextended GP better than PTUR and Reciproc files [42].

The complete removal of the filling material was only achieved in three curved roots re-shaped with a combination of manual K and H-files [12]. In the other studies which used manual files, it was not achieved in any tooth [2,4,15,25,26,34,36,39,55,58,61].

Some authors observed that, in teeth instrumented with rotary files, more dentine is lost, but fewer canal transportation occurs than with the use of hand files [58]; meanwhile, others consider the use of PTUR files to be less invasive in root dentine than the use of H-files [63]. These two articles use very similar evaluation methods, so the differences may be due to the use of solvent, because in one article it is used [63] but in the other it is not [58]. Other authors such as Aarthi et al. [70] found no significant differences, although they found that PTUR produced more dentine damage than H-files. Parallelly, H-files produced more dentine damage than RB, with no significant differences.

It has also been observed that the use of rotary files considerably reduces treatment times [4,8,16,18,21,31,34,36,39,47,55,56,61,66], which is especially the case for the PTUR file [25,47,55].

Regarding apical extrusion of debris, it has been reported that it is higher when using hand files [36,41,56] and that the occurrence of dentine defects or fissures is higher when using rotary files [31,44,66].

### 4.2. Continuous-Rotation Rotary Files

Among the rotary instruments studied, no system completely removed the remaining filling material [3,4,5,13,15,25,34,39,40,47,49,58,61,62].

Many authors agree that PTUR files are the most effective in the removal of filling material, being superior to RaCe [2,38], ProFile [4], GPR [34], MtwoR [27], and R-Endo [61], but less effective than Neoendo files [61]. However, according to Hasija et al. [52], MtwoR removes more filling material than PTUR. It is worth mentioning that this author used solvent, while others did not [27]). For Reddy et al. [15], R-Endo performs better than PTUR. It should be highlighted that this author did not use solvent [15], while others used Xylene [61]. Parallelly, Akshay et al. [5] demonstrated that TrN also works significantly better in removing material inside the canal. Hyun et al. [65] found no significant differences between PTUR and HFR, and Karunakar et al. [73] found no significant differences between HFR and NeoEndoR. Akhavan et al. [19] found no significant differences between MtwoR and D-RaceR, and Agrawal et al. [49] described a higher effectiveness of MtwoR versus R-Endo in the removal of filling material. Sameh and Omaia [3], when comparing Re-Endo with Rogin, found that Rogin cleaned the filling debris significantly better. In addition, the largest amount of remnant material remains in the apical third [3,15,18,47].

If the instrumentation time during retreatment is analyzed, it can be observed that PTUR files are faster than R-Endo [55], EF-X3 [62], TF [13], D-RaCe [47], GPR [34], ProFile [25], and MtwoR [18,27,34,55]. However, Marfisi et al. [13] found MtwoR files to be faster than PTURs, although this difference was not significant. In this case, we should highlight that this article [13] presents a moderate risk of bias, while others [55] that reach different results present a low risk of bias. Akshay et al. [5] concluded that RaCe files removed canal filling material faster than PTURs and Hyun et al. [65] concluded that HFR takes less time than PTURs to remove the filling from the canal.

Uezu et al. [14], when comparing PTUR files with PTU files, found PTU to be significantly faster in removing root canal filling material. In the study by Çelik Ünal et al. [12] on teeth with curved roots, PTUR and R-Endo were less effective in removing filling material compared to ProFile, and also required more working time.

Several authors agree on the high number of dentinal defects produced by PTUR files: more than XP-ES + XP-EF [57], R-Endo [44], PTN [43], MtwoR [43], TFA [43], EF-XRR [64], and S-RS3 [68,75,76]. In this manner, Kulkarni et al. [51] found that PTUR leaves a much lower dentine thickness than D-RaCe and MtwoR after root canal retreatment. However, Ali et al. [58] noted a greater loss of dentine with the R-Endo system than with PTUR. In this case, the article by Ali et al. [58] presents a low risk of bias, while that of Jain, Nikhil, and Bansal [44] presents a high risk of bias.

Comparing GPR files with Re-Endo, the former showed more crack formation [66]. Varghese et al. [7] found that the Edge File XR produced fewer dentine defects compared to MtwoR and PTUR, with significant differences between them.

When looking at the effect of HFR versus S-RS3 in retreatment, there is no significant difference in the dentine removed in the distal area, but there is a significant difference in the mesial area [77]. When comparing S-RS3 with S-RS3B and S-REB, the latter shows significantly higher dentine removal compared to S-RS3 and S-RS3B, being similar in these two [79].

Kim, Chang, and Oh [9] compared D-RaCe, HFR, and MtwoR, and observed that the maximum torque was found in HFR, followed by MtwoR and D-Race; the highest specific density was found in MtwoR files, followed by HFR and D-Race.

Regarding the amount of material extruded to the periapical area, PTUR produced more extrusion than Re-Endo [45], TrN [5], RaCe [5], and MtwoR [89]; meanwhile, MtwoR produced more extrusion than R-Endo [21].

Regarding procedural errors, fractures (5 PTUR and 2 R-Endo files) were recorded in the treatment of 56 curved molar roots [12], as well as the fracture of one MtwoR and two TF files in a sample of 90 single-rooted teeth [13].

### 4.3. Reciprocating Files

Only one doctoral thesis was found that compares purely reciprocating files and concludes that Reciproc can be a good choice for re-treatment because it produces less apical extrusion than WO files [90].

### 4.4. Rotary Files: Continuous Motion vs. Reciprocating Motion

When comparing continuous-rotation files with reciprocating files, it is observed that apical extrusion is lower when continuously rotating files are used. Some studies found statistically significant differences [41], while others did not [67]. The difference in significance may be due to the fact that the study by Abdelnaby, Ibrahim, and ElBackly [67] presented a low risk of bias, while the study by Kayahan et al. [41] did not specify the methodology in such detail and presented a moderate risk of bias. When open apices are present, extrusion is lower with reciprocating files [56].

Regarding the amount of material remaining inside the canal after retreatment, it was lower with WO compared to PTUR [50], lower with WOG than with MtwoR [53], and lower with RB than with HF EDM and PTUR [67], i.e., it was lower in reciprocating files, although this was without significant differences. However, neither system was able to completely remove the remnant filling from inside the canal [53,67].

Regarding working time, for Topçuoğlu et al. [56], it was shorter when they used reciprocating files; meanwhile, for Abboud et al. [71], the time was longer when they used WOG files than then they used PTUR. This difference could be because Abboud et al. [71] measured T1, T2, and Tt, while Topçuoğlu et al. [56] measured the time required to remove the root canal filling.

While some studies consider RB to produce fewer dentinal defects, although without significant differences [70], others have associated it with increased defect formation in the coronal and middle thirds [32]. In both studies, the evaluation method was visual, and the risk of bias was moderate.

### 4.5. Influence of Supplementary Files

Several articles studied the effect of different retreatment files when used in combination with other supplementary files in order to improve the outcome of the procedure [6,20,22,23,29,33,37,54,69]. In this manner, Pawar et al. [37] compared the use of PTUR files and their association with PTN, WO, and SAF, observing that PTUR used with SAF produces less extrusion than when used with PTN or WO. Voet et al. [23] also noted that the use of PTUR improved when used in combination with SAF. SAF, used as a supplementary file, also improved the result of the R-Endo file in terms of canal cleaning, without completely removing all the residual root filling material [29]. The same occurred with ProFile [22]. Marques da Silva et al. [20] compared the use of PTUR, D-RaceR, and MtwoR alone and with additional files and observed that there were no significant differences between the study groups. Furthermore, they noted that all the tested teeth had remaining filling material in the canal except two cases instrumented with PTUR and additional files.

Tsenova-Ilieva et al. [69] observed that, with the use of supplementary files, more remaining filling material was removed, but without significant differences. Mutar and Al-Zaka [54] studied the use of D-RaCe alone and in combination with RB, PTN, and PTG, and found no significant differences between the study groups. In the study by Shaheen, Elhelbawy, and Sherif [6], the use of PTUR alone was compared with its use together with WOG, XP-EF or TrN; the WOG file produced the most apical extrusion, followed by TrN and finally XP-EF. No relationship was observed between apical permeability and extrusion. For Çiçek et al. [33] and Pawar et al. [37], significantly more apical extrusion occurred when complementary files were used [33,37].

### 4.6. Limitations and Future Perspectives

Although studies have been gradually implementing similar study methods, there is no consensus in the procedures used to evaluate the effectiveness of files in root canal retreatment, in a way that no direct comparisons can be made between different studies. Additionally, there is also no uniformity in sample selection (size and root typology). This methodological heterogeneity hindered the performance of a robust quantitative synthesis or meta-analysis.

Specific replicable protocols for the evaluation of the efficacy of endodontic files in root canal retreatment should be developed in order to allow a homogenous comparison between studies to be achieved.

## 5. Conclusions

Despite the heterogeneity among the methodology of the included studies, the following conclusions can be drawn from their results: Manual files produce more apical extrusion than rotary files. PTUR files are the most-studied endodontic files, being the file system that leaves the least residual filling material in the canal, takes the least time during retreatment, and removes the greatest amount of dentine. However, no file system achieved a complete removal of the root canal filling material. No consistent pattern emerged across studies when comparing rotary files with continuous and reciprocating rotation in terms of removal of filling material, time required for de-obturation, dentine damage produced, or apical extrusion.

## Figures and Tables

**Table 1:** Search strategy and findings per database.

Database	Search Strategy	Findings
Medline	#1 “retreatment files”	113
	#2 (cleaning OR shaping OR extrusion OR debris OR dentin)	796,240
	#1 AND #2	67
Scopus	#1 ALL (“retreatment files”)	163
	#2 ALL (cleaning OR shaping OR extrusion OR debris OR dentin)	754,624
	#1 AND #2	69
Embase	#1 “retreatment files”	98
	#2 (cleaning OR shaping OR extrusion OR debris OR dentin)	234,462
	#1 AND #2	43
Web of Science	#1 TS = (“retreatment files”)	153
	#2 TS = (cleaning OR shaping OR extrusion OR debris OR dentin)	5,591,577
	#1 AND #2	95
SciELO	#1 “retreatment files”	4
	#2 (cleaning OR shaping OR extrusion OR debris OR dentin)	6567
	#1 AND #2	0
Cochrane	#1 “retreatment files”	13
	#2 (cleaning OR shaping OR extrusion OR debris OR dentin)	28,464
	#1 AND #2	5

**Table 2:** Summary of the articles selected for the systematic review: procedure and risk of bias.

Author, Year	Sample	Previous Root Canal Treatment	Studied Files	Solvent	Studied Parameters	Risk of Bias
Giuliani, Cocchetti y Pagavino (2008) [4]	*n* = 42; Anterior teeth, single root	It: 30/0.06 (ProFile) O: Continuous wave technique (Obtura II)	PTUR ProFile H + K	Endosolv E	- LC: OSMx40	Moderate
Somma et al. (2008) [8]	*n* = 90; PM, straight (<5° curvature)	It: 40/0.04 (MTwo) O: - *n* = 30 GP + Kerr Pulp Canal Sealer - *n* = 30 Resilon Real Seal - *n* = 30 GP + EndoRez	PTUR MtwoR H	Chloroform	- EA: Scoring system 0–3 - AI: Perforation/blockage count - LC: OSMx8, x16, x32/SEM x50, x150, x300, x600 - T: seconds (s)	Moderate
Çelik Ünal et al. (2009) [12]	*n* = 56; M, 20–42° curvature	It: 30/0.06 (ProFile) O: Continuous wave technique (Obtura II + AHPlus)	ProFile R-Endo PTUR K + H	Eucalyptol	- EA: Score 0–2 - AI: Count - LC: Area (%) of the canal covered by residual material (periapical rx + AutoCAD 2000 software) - T: s	Moderate
Marfisi et al. (2010) [13]	*n* = 90; single roots (30 ILs, 30C, 30 PM)	It: Crown-down LAM30 O: - *n* = 45 GP + AHPlus - *n* = 30 resilon + Real Seal dual	PTUR MtwoR TF	-	- LC: CBCT + iCAT software to calculate area (%) covered by filling material Visual scoring 1–5 - T: s	Moderate
Uezu et al. (2010) [14]	*n* = 30; PM, straight	It: 30 (F3-PTU) O: Single cone + Nrickert (ZOE)	PTU PTUR	-	- EA: Weighing - AI: Fracture count - T: s	High
Reddy et al. (2011) [15]	*n* = 90; PM mb, (40–45° curvature)	It: 25/0.07 (Mtwo) O: continuous wave technique. GP + sealer: - *n* = 45 AHPlus - *n* = 45 ZOE	H PTUR R-Endo	-	- LC: OSMx40. Area (%) of residual material with AutoCAD 2007 software	Moderate
Shemesh et al. (2011) [16]	*n* = 200; PM mb	It: 40 (F4-PTU) O: lateral condensation. GP + AHPlus	PTUR H	Chloroform	- DD: OSMx12. Classification: “with defects,” “without defects,” or “fracture” - T: minutes (min)	Moderate
Siotia, Acharya y Gupta (2011) [17]	*n* = 45; single root PM	It: 25 (F2-PTU) O: - *n* = 15 GP + AHPlus - *n* = 15 GP + ZOE - *n* = 15 GuttaFlow	PTUR	-	- LC: OSMx10 Area (%) of residual material with AutoCAD 2004 software	Moderate
Yilmaz, Karapinar and Ozcelik (2011) [18]	*n* = 63; single root, curved (<10°)	It: 35/0.04 (Mtwo) O: - *n* = 21 BeeFill + 2 seal - *n* = 21 BeeFill + AH26 - *n* = 21 lateral condensation GP + AH	MtwoR PTUR H	-	- LC: OSMx8, x16, x32 Area (%) of remaining material using COMEF 4.3 software - T: Stopwatch (s)	Moderate
Akhavan et al. (2012) [19]	*n* = 60; first M mb	It: step-back LAM60 O: lateral condensation GP + AHPlus	MtwoR D-RaCeR	Chloroform (half the sample)	- LC: OSMx16 and AutoCAD 2009 software	High
Marques da Silva et al. (2012) [20]	*n* = 90; single root PM	It: Step-down LAM30 O: Hybrid Tanger technique. GP + AHPlus	PTUR D-RaceR MtwoR	-	- LC: High-resolution scanner + AutoCAD 2004 to calculate % residual obturation material	Moderate
Mollo et al. (2012) [21]	*n* = 60; single root mx anterior teeth	It: 35 (PTU-F3) O: continuous wave technique. SystemB + ObturaII + Pulp Canal Sealer	MtwoR R-Endo K	Chloroform (only with K files)	- EA: Visually (yes/no) - AI: Fracture count - LC: rx + AutoCAD 2004. Calculation of area (%) with residual material - T: TT	Low
Solomonov et al. (2012) [22]	*n* = 28; M mb	It: 25 (PTU-F2) O: lateral condensation. GP + AHPlus.	PTUR + PTU ProFile + SAF	Chloroform	- LC: MicroCT before, after obturation, and after retreatment. Volume of remaining material (mm^3^) - T: Stopwatch (min)	Low
Voet et al. (2012) [23]	*n* = 33; 1st M mx, mesiobuccal root, 25–35° curvature	It: 30 (PTU-F3) O: GP30 + AH26	PTUR + H PTUR + H+SAF	-	- LC: OSMx40. Area (%) of residual material and visual score 1–5	Moderate
Reddy et al. (2013) [24]	*n* = 60; Anterior teeth, single root	It: step-back LAM30 O: lateral condensation. GP + AHPlus	H PTUR	Xylene (*n* = 30)	- LC: OSMx20. Visual score 1–4 - T: Stopwatch	Moderate
Shivanand et al. (2013) [25]	*n* = 60; PM mb	It: step-back LAM30 O: thermoplastic GP + AHPlus	PTUR ProFile H	Eucalyptol	- EA: Visually - AI: Fracture count - LC: OSMx70 and visual scoring [81] - T: s	Moderate
Yadav et al. (2013) [26]	*n* = 30; PM mb with 1 canal	It: step-back LAM30 O: lateral condensation. GP + AHPlus	PTUR MtwoR H	Xylene	- EA: visual score - AI: count of fractured files - LC: CBCT. Volume (mm^3^) of residual material.	Moderate
Chandrasekar et al. (2014) [2]	*n* = 40; ICs mx, straight	It: step-back LAM30 O: lateral condensation. GP + ZOE	PTUR RaCe K3 H	Orange oil	- EA y LC: spiral CT and Syngo software. Volume (cm^3^ and %) of removed obturation material.	Moderate
İriboz y Sazak Öveçoğlu (2014) [27]	*n* = 160; Anterior teeth mx with 1 canal	It: 30 - *n* = 80 PTU (until F3) - *n* = 80 Mtwo (until 30/0.05) - O: lateral condensation *n* = 20 Resilon + Epiphany - *n* = 20 GP + Epiphany - *n* = 20 GP + AHPlus - *n* = 20 GP + Kerr Pulp Canal Sealer (PCS)	PTUR ( + PTU) MtwoR ( + Mtwo)	-	- AI: Count of fractured files - LC: SEM x500 and x1500. Visual score (%) [82] - T: T1, T2, TT (including instrument changes and irrigation)	Moderate
Keles et al. (2014) [28]	*n* = 20; PM mb with 1 canal	It: 25/0.06 (Revo-S) + K45 O: GP + AHPlus - *n* = 10 lateral condensation - *n* = 10 continuous wave technique	R-Endo SAF	-	- LC: MicroCT and NRecon software pre- and post-retreatment. Volume (%) of remaining obturation material.	Moderate
Keleş et al. (2014) [29]	*n* = 48; PM mx, straight, with 1 canal	It: 40/0.06 (Revo-S) + K45 O: continuous wave technique + AHPlus	R-Endo R-Endo + SAF	-	- LC: SEM x2000 and x100. Visual score scale 1–3	Moderate
Topçuoǧlu et al. (2014) [30]	*n* = 80; PM mb	It: 35/0.06 (Revo-S) O: single cone + AHPlus	PTUR ( + F4-PTU) MTwoR ( + Mtwo40/0.04) H	Eucalyptol	- DD: OSM x20. Visual evaluation for presence of cracks or fractures	Moderate
Topçuoğlu et al. (2014) [31]	*n* = 180; PM mb	It: step-back LAM40 O: passive technique with GAM40 and additional GP cones + AHPlus	MTwoR D-RaCe R-Endo H	Chloroform	- DD: OSMx20. Visual assessment of defects [83] - T: s (excluding instrument change and irrigation)	Low
Üstün et al. (2015) [32]	*n* = 120; PM mb	It: step-back LAM35 O: passive technique with GAM35 and additional GP cones + AHPlus	PTUR ( + F4-PTU) Reciproc	Eucalyptol	- DD: OSMx20. Visual assessment of defects [83]	Moderate
Çiçek et al. (2016) [33]	*n* = 60; PM mb with 1 canal	It: 25 (PTU-F3) O: lateral condensation + AHPlus	MtwoR MtwoR + Mtwo30 PTUR PTUR + F2-PTU	-	- EA: Weighing with microbalance	Moderate
Joseph et al. (2016) [34]	*n* = 60; PM mb with 1 canal	It: step-back LAM30 O: hybrid technique, lateral condensation GP + AHPlus	PTUR MTwoR GPR H	Eucalyptol (GPR and H groups)	- LC: OSMx12.5 and Image-pro Express software. Calculation of area (% and mm^2^) of remaining material - T: Stopwatch (s)	Moderate
Kanaparthy (2016) [35]	*n* = 60; PM, straight	It: - *n* = 20 step-back LAM20 - *n* = 20 20/0.07 PTU - *n* = 20 20/0.06 Mtwo - O: lateral condensation *n* = 30 ZOE - *n* = 30 AHPlus	H PTUR MtwoR	-	- LC: OSMx4. Visual evaluation [8]	Moderate
Kasam and Mariswamy (2016) [36]	*n* = 48; PM mb with 1 canal, <10° curvature	It: step-back LAM40 O: lateral condensation GP + ZOE	H H-Safe-sided PTUR US	Xylene	- EA: Microbalance (g) - LC: OSMx20 and AutoCAD software. Volume calculation (% and mm^3^) of residual material - T: s	Moderate
Pawar et al. (2016) [37]	*n* = 80; PM mb with 1 canal	It: 30 (PTU-F3) O: lateral condensation. GP F3 + accessory GP + AHPlus	PTUR PTUR + PTN PTUR + WO PTUR + SAF	Endosolv R	- EA: Weighing of extruded residue	Moderate
Preetam et al. (2016) [38]	*n* = 30; PM mb with 1 straight canal	It: PTU O: Thermoplastic GP. Obtura II system	PTUR RaCe H	-	- LC: rx visual opacity score of residues (0–3)	Moderate
Monardes et al. (2016) [39]	*n* = 45; Canals with 5–20° curvature	It: 25/0.06 (Mtwo) + 40 K O: Lateral condensation. GP + Tubliseal (ZOE)	PTU PTUR H + K	-	- LC: rx visual score (0–3) - T: Stopwatch (s)	Moderate
Das et al., (2017) [40]	*n* = 60; PM mb with 1 straight canal	It: Step-back LAM25 O: Lateral condensation. GP + AHPlus	PTUR MtwoR R-Endo	RC Solve (*n* = 30)	- LC: OSMx16 and DX-312 software. Area calculation of residual material (mm^3^)	Moderate
Kayahan et al. (2017) [41]	*n* = 45; ISs and C with <10° curvature	It: 50 (PTU-F5) O: Lateral condensation. GP + AHPlus	PTUR + PTU (until F5) Reciproc (R25, R40, R50) K	-	- EA: weighing (g)	Moderate
Kesim et al. (2017) [42]	*n* = 80; PM mb with <10° curvature	It: 35/0.06 (Revo-S) O: Lateral condensation. GP + MM seal (resin-based)	PTUR MtwoR Reciproc H	-	- Overextended GP removal: OSMx12.5. Visual assessment: “successful”/”failed” GP removal	Moderate
Özyürek et al. (2017) [43]	*n* = 120; PM mb, straight	It: Crown-down with K files up to 40/0.02 O: Continuous wave technique. GAM40/0.02 + AHPlus	PTUR MtwoR PTN TFA H	-	- DD: OSM. Visual assessment of appearance, presence, or propagation of cracks	Low
Jain, Nikhil and Bansal (2018) [44]	*n* = 45; PM mb with 1 canal	It: 40 (PTU-F4) O: GP + AHPlus	PTUR + F4-PTU R-Endo + 40 Hero Shaper H	Xylene (H-files only)	- DD: MicroCT. Crack and fracture count	High
Jena et al. (2018) [45]	*n* = 30; PM mb with 1 canal, <10° curvature	It: 30 (PTU-F3) O: Vertical condensation. GP + AHPlus	PTUR Re-Endo	RC Solve	- EA: weighing with microbalance (g)	Moderate
Kakoura and Pantelidou (2018) [46]	*n* = 60; C with 1 canal and <20° curvature	It: 40/0.04 (Sendoline) O: single cone - *n* = 20 GP + AH26 - *n* = 20 GP + TotalFill - *n* = 20 GP + BioRoot	PTUR + K	-	- LC: SEMx100 and x1000 and energy-dispersive spectroscopy. Visual score 1–4 [84] for residual material	Low
Raj et al. (2018) [47]	*n* = 63; ICs mx with 1 canal	It: step-back LAM30 O: lateral condensation. GP + AHPlus	D-RaCeR PTUR H	RC Solve (H-files only)	- LC: SEMx1000 and Motic Image Plus software. Clean area (%) calculation - T: TT	Moderate
Rödig et al. (2018) [48]	*n* = 60; Curved roots of M	It: 30/0.04 (ProFile) O: AHPlus - *n* = 20 Thermafil - *n* = 20 Guttacore - *n* = 20 Continuous wave technique	PTUR	-	- AI: Count of plugs, fractures, perforations - LC: MicroCT and NRecon software. Residual volume calculation (mm^3^) of obturation material - DD: Residual dentinal volume calculation (mm^3^) - T: T1, T2, TT (excluding instrument changes and irrigation)	Moderate
Agrawal et al. (2019) [49]	*n* = 60; PM with 1 canal, <10° curvature	It: 30 (PTU-F3) O: lateral condensation. GP + ZOE	US R-Endo MtwoR	-	- LC: OSMx40 and Image-pro Express software for residual material evaluation	Moderate
Ali et al. (2019) [50]	*n* = 60; ISs mx with <10° curvature	It: 30/0.06 (Neoendo Flex 30) O: Continuous wave technique (Fast-Pack) + AHPlus	H PTUR GPR WO	-	- LC: OSMx8 and COMEF software. Area (%) of residual material	Low
Kulkarni et al. (2019) [51]	*n* = 45; Mx 1st M, mesiobuccal roots	It: 25 (PTUR-F2) O: lateral condensation. GAM25 + AHPlus	PTUR + PTU MtwoR + Mtwo D-Race + IRace	RC Solve	- DD: CBCT to assess residual dentin thickness (mm) by thirds	High
Hasija et al. (2020) [52]	*n* = 90; ICs and C with <10° curvature	It: 40/0.04 (Mtwo) O: lateral condensation. GP + Bioseal (resinoso)	H PTUR MtwoR H + láser neodinio	Endosolv-R (*n* = 40)	- LC: SEMx1000. Grayscale-based evaluation	Moderate
Maruster et al. (2020) [53]	*n* = 12; Resin blocks	It: 25 (PTN-X2) O: AdSeal + - *n* = 6 GuttaCore - *n* = 6 Guttafusion	WOG MtwoR	-	- LC: Visual score 0–4 - DD: ImageJ. Calculation of removed wall volume - T: T1 and T2 (min and s)	High
Mutar y Al-Zaka (2020) [54]	*n* = 60; PMs mb with straight canals	It: 40 (PTN-X4) O: GP X4 + GuttaFlow2	D-RaCe D-RaCe + RB D-RaCe + PTN D-RaCe + PTG	-	- LC: OSMx12.5 and Adobe Photoshop C to calculate % of remaining material	Moderate
Purba et al. (2020) [55]	*n* = 70; PMs mb with 1 straight canal	It: 25 (PTU-F2) O: warm lateral condensation. GP F2 + AHPlus	H PTUR MtwoR R-Endo	Wonder Orange (*n* = 40)	- LC: Longitudinal section. OSMx20 and residual material calculation (%) [85] - T: T1, T2, Tt (min)	Low
Topçuoğlu et al. (2020) [56]	*n* = 120; PMs mb with 1 canal, <10° curvature	It: 40/0.06 (Revo-S) O: lateral condensation. GP + AdSeal	H PTUR D-Race Reciproc	-	- EA: weighing - T: s	Low
AbuMostafa, Almoqayyad y Mohammad (2021) [57]	*n* = 60; PMs with 1 straight canal	It: 40 (PTN-X4) O: lateral condensation. GP + AHPlus	PTUR XP-ES + XP-EFR	Gutasolv	- DD: Horizontal section. OSMx50 and visual evaluation of defects [83]	Low
Ali et al. (2021) [58]	*n* = 60; ILs mx with 20–35° curvature	It: 40/0.04 (Neoniti-A1) O: lateral condensation. Guttaflow.	PTUR R-Endo H	-	- LC: CBCT. Area (%) of remaining material - DD: Canal transportation assessment (none, mesial, distal)	Low
Colmenar et al. (2021) [59]	*n* = 10; Anterior teeth with 1 canal	It: 50 (PTN-X5) O: single cone - *n* = 5 GP + AHPlus - *n* = 5 GP + Endosequence	PTUR	-	- LC: MicroCT and CTAn software. Calculation of volume (%) of removed obturation material	Moderate
Eid, Maksoud and Elsewify (2021) [60]	*n* = 40; ISs mb with <20° curvature	It: 30 (PTN-X3) O: continuous wave technique. TotalFill BC sealer.	GPR + XP-EFR GPR + H	-	- LC: MicroCT and NRecon software. Residual material calculation (mm^3^)	Moderate
Muraleedhar et al. (2021) [61]	*n* = 48; PMs, single-rooted	It: step-back LAM30 O: lateral condensation. GP + AHPlus	H PTUR R-Endo NeoendoR	Xylene	- LC: Longitudinal section. OSMx12.5 and Image Pro software. Residual material calculation (%) - T: Stopwatch	Moderate
Özlek and Gündüz (2021) [62]	*n* = 40; PMs with 1 canal, <10° curvature	It: 25 (PTU-F2) O: single cone GP + MTA fillapex	PTUR PTN EF-XRR EF-X3	-	- LC: MicroCT and TC Evaluation Program software. Volume of residual material (mm^3^ and %) - T: T1, T2 excluding instrument changes and irrigation (s)	Moderate
Subramanian et al. (2021) [63]	*n* = 20; ICs mx with 1 canal	It: 30 (PTN-X3) O: GP X3 + AHPlus	H PTUR	Xylene (*n* = 10)	- DD: CBCT. Measurement of residual dentin thickness (mm) in axial cuts	Moderate
Das et al. (2022) [64]	*n* = 80; PMs mb with 1 canal	It: 30 (PTU-F3) O: GP F3 + resin-based sealer	EF-XRR PTUR MtwoR	RC Solve	- DD: Cross sections. OSMx20. Visual evaluation of defect appearance [86]	Moderate
Hyun et al. (2022) [65]	*n* = 40; PMs with 1 oval canal and 10–20° curvature	It: 40/0.06 (PTU-F4) O: GP + Ceraseal - *n* = 20 GP4% - *n* = 20 GP6%	PTUR HFR	-	- EA: Weighing (mg) - LC: Longitudinal section. OSMx20 and visual score [8] - T: T1 (s) without instrument change	Moderate
Sameh and Omaia (2022) [3]	*n* = 45; PMs mb	It: 30 (PTN-X3) O: GP X3 + Adseal	US Re-Endo RoginR	-	- LC: Longitudinal section. OSMx15 and ImageJ software. Residual material area	Moderate
Shaheen, Elhelbawy and Sherif (2022) [6]	*n* = 80; PMs with 1 canal and <20° curvature	It: 30 (PTU-F3) O: GP F3 + Endosequence BC	PTUR PTUR + WOG PTUR + XP-EFR PTUR + TrN	-	- EA: analytical balance (g)	Moderate
Tejaswi et al. (2022) [66]	*n* = 60; 1st M mx	It: step-back LAM35 O: lateral condesation. GP + AHPlus	GPR Re-Endo H	-	- DD: Horizontal sections. SEMx50, x100, x3000. Dentinal crack count - LC: Presence/absence of GP remnants - T: Tt. Stopwatch (s)	Moderate
Abdelnaby, Ibrahim and ElBackly (2023) [67]	*n* = 36; 1st M mb with 2 separate mesial canals	It: 25 (PTN-X2) O: lateral condensation. GP X2 + Adseal + accessory GP 20/0.02	RB HF EDM PTUR + PTN	-	- EA: Microbalance (g) - AI: Fracture count - LC: CBCT. Residual material volume calculation (mm^3^, %)	Low
Akshay et al. (2023) [5]	*n* = 45; M mb with 10–20° curvature in mesiobuccal root	It: 25/0.04 (RaCe) O: GAM25/0.04 + BioC Sealer + accessory GP	TrN PTUR RaCe	-	- EA: Visual score 0–2 - LC: CBCT and Romexis software. Residual and removed material volume calculation (%) - T: T1, T2, TT (no instrument change or irrigation). Stopwatch (s)	High
Baig et al. (2023) [1]	*n* = 120; PMs mb with 1 straight canal	It: step-back LAM30 O: lateral condensation. GP + AHPlus	H D-RaCe MtwoR	RC Solve (*n* = 60)	- LC: Longitudinal section. OSMx20 and visual score 1–4 [87]	Moderate
Kim, Chang and Oh (2023) [9]	*n* = 45; Resin blocks with J-shaped canals	It: 30 (PTN-X3) O: cono único GP + AHPlus	D-RaCe HFR MtwoR	-	- Buckling resistance: compression of a file in a dimple - LC: OSMx20 and ImageJ software. Area (%) of residual material	Moderate
Rama Sowmya et al. (2023) [68]	*n* = 60; PMs mb with <15° curvature	It: step-back LAM40 O: lateral condensation. GP + AHPlus	PTUR S-RS3	-	- DD: CBCT. Measurement of residual dentin thickness	Moderate
Tsenova-Ilieva et al. (2023) [69]	*n* = 12; ISs mb with 1 straight canal (<5° curvature)	It: 30/0.04 (XP-ES) + Glyde (EDTA) O: continuous wave technique. GP + AHPlus	MtwoR MtwoR + XP-EFR	-	- AI: Count of broken files and errors - LC: 3 MicroCTs, CB Studio MAX software. Residual volume calculation (mm^3^)	Low
Varghese et al. (2023) [7]	*n* = 60; PMs mb with 1 canal	It: 30 step-back + PTU-F3 O: GP6% + sealer	MTwoR PTUR EF-XRR	-	- DD: MicroCT. Lesioned radicular dentin volume (mm^3^)	High
Aarthi et al. (2024) [70]	*n* = 60; ICs mx	It: 30 (PTU-F3) O: GP F3 + AHPlus	H PTUR RB	-	- DD: Horizontal section. OSMx40 and visual score [83]	Moderate
Abboud et al. (2024) [71]	*n* = 45; PMs with <5–10° curvature	It: 25/0.06 (AF Gold) O: lateral condensation. GP + AdSeal	PTUR WOG	-	- T: T1, T2, TT. Stopwatch (s)	Low
Buyuksungur et al. (2024) [72]	*n* = 27; M mb, mesiobuccal roots with 30° curvature	It: 25 (PTG-F2) O: GP F2 + sealer - *n* = 9 AHPlus - *n* = 9 Well Root-ST - *n* = 9 AHPlus Bioceramic Sealer	PTUR	-	- LC: 3 MicroCTs and CATn and NRecon software. Residual volume calculation (%)	Low
Karunakar et al. (2024) [73]	*n* = 30; Teeth with 1 canal	It: 25 (PTU-F2) O: GP F2 + AHPlus	NeoEndoR HFR H	-	- LC: Longitudinal section. OSMx20 and visual score [85]	Moderate
Nour, Elgendy and Bayoumi (2024) [74]	*n* = 45; PMs mb with 1 canal	It: 40 (PTU-F4) O: GP + sealer - *n* = 14 single cone - *n* = 14 continuous wave technique - *n* = 14 lateral condensation	PTUR	-	- EA: Microbalance weighing [88]	Moderate
Sairaman et al. (2024) [75]	*n* = 40; Anterior teeth with 1 canal and <5° curvature	It: 30 (PTG-F3) O: GP F3 + AHPlus	S-RS3 PTUR	-	- DD: MicroCT and NRecon software. Canal transportation calculation	Moderate
Sairaman et al. (2024) [76]	*n* = 60; Teeth with 1 straight canal (<5° curvature)	It: 30/0.06 (ProFit S3-PF3) O: single cone. GP PF3 + AHPlus	S-RS3 PTUR	-	- DD: MicroCT and NRecon software. Canal transportation calculation	Low
Sankar et al. (2024) [77]	*n* = 60; PMs mb with 1 canal and <15° curvature	It: step-back LAM40 O: lateral condensation. GP + AHPlus	HFR S-RS3	-	- DD: CBCT and Imaging software. Dentin thickness calculation	Moderate
Singh et al. (2024) [78]	*n* = 60; 1st M mx with <15° curvature in palatal root	It: step-back LAM40 O: lateral condensation. GP + AHPlus	NeoEndoR R-Endo K + H	-	- LC: CBCT and Image I software. Removed volume calculation - T: T1, T2, TT. Stopwatch, min (including instrument changes and irrigation)	High
Suresh et al. (2024) [79]	*n* = 45; PMs mb with 1 canal	It: 25/0.06 (ProFit S3-PF2) O: GP25/0.06 + AHPlus	S-RS3 S-RS3B S-REB	-	- DD: NanoCT & NRecon and CTAn software. Remaining dentin depth calculation (%) and fissure appearance.	Low
Yadav et al. (2024) [80]	*n* = 60; PMs with 1 canal and <30° curvature	It: 30/0.06 (NiTi) O: - *n* = 20 RealSeal Resilon - *n* = 20 GuttaFlow2 - *n* = 20 Activ GP	D-RaCe	-	- LC: Mesiodistal and buccolingual digital rx and Adobe Photoshop software. Area with residual material (%) - T: TT (no instrument changes or irrigation). Stopwatch (s)	High

ISs: incisors; ICs: central incisors; ILs: lateral incisors; Cs: canines; PMs: premolars; Ms: molars; mx: maxillary; mb: mandibular; LAM: master apical file; GAM: master apical gutta-percha; US: ultrasonic tip; EA: apical extrusion; AI: intraoperative accidents; LC: canal cleaning; DD: dentinal structural damage; T: time; It: instrumentation; O: obturation; GP: gutta-percha; ZOE: zinc oxide eugenol–based sealer; s: second; min: minute; OSM: stereomicroscope; SEM: scanning electron microscope; rx: radiograph; T1: time to reach working length; T2: time to completely remove obturation material; Tt: T1 + T2.

**Table 3:** Studied files.

File	Rotation	Primary File	Accessory File
ProTaper Universal Retreatment (PTUR; Dentsply-Maillefer, Ballaigues, Switzerland)	Continuous	59	
ProFile (Dentsply-Maillefer, Ballaigues, Switzerland)	Continuous	4	
H-file	Manual	34	1
K file	Manual	6	
Mtwo R (Seden & Martina, Padua, Italy)	Continuous	26	
R-Endo (Micro-Mega, Besançon, France)	Continuous	13	
Twisted Files (TF, Sybron Dental Specialties, Orange, USA)	Continuous	1	
Twisted File Adaptive (TFA; SybronEndo, Orange, USA)	Continuous	1	
ProTaper Universal (PTU; Dentsply-Maillefer, Ballaigues, Switzerland)	Continuous	2	8
D-RaCe (FKG Dentaire, La Chaux-de-Fonds, Switzerland)	Continuous	10	
Self-Adjusting File (SAF; ReDent, Ra’anana, Israel)	Reciprocating	3	4
RaCe (FKG Dentaire, La Chaux-de-Fonds, Switzerland)	Continuous	3	
K3 (SybronEndo, Orange, CA, USA)	Continuous	1	
Reciproc (VDW, Munich, Germany)	Reciprocating	4	
Hedstrom safe-sided	Manual	1	
ProTaper Next (PTN; Dentsply-Maillefer, Ballaigues, Switzerland)	Continuous	2	3
Endostar RE Re-Endo (Re-Endo; Endostar, Poldent Co., Warsaw, Poland)	Continuous	3	
NRT-GPR (GPR; Mani Inc., Tokyo, Japan)	Continuous	4	
Wave One (WO; Dentsply-Maillefer, Ballaigues, Switzerland)	Reciprocating	1	1
Wave One Gold (WOG; Dentsply-Maillefer, Ballaigues, Switzerland)	Reciprocating	2	1
XP-endo Shaper (XP-ES; FKG Dentaire, La Chaux-de-Fonds, Switzerland)	Continuous	1	
XP-endo Finisher (XP-EFR; FKG Dentaire, La Chaux-de-Fonds, Switzerland)	Continuous	1	3
NeoEndo Retreatment (NeoendoR; Orikam Healthcare, India)	Continuous	3	
EdgeFile XR Retreatment (EF-XRR; EdgeEndo, USA)	Continuous	3	
EdgeFile X3 (EF-X3; EdgeEndo, Albuquerque, USA)	Continuous	1	
HyFlex Remover (HFR; Coltene/Whaledent AG, Altstätten, Switzerland)	Continuous	4	
Rogin (RoginR; Shenzhen Rogin Medical Co., China)	Continuous	1	
Reciproc Blue (RB; VDW, Munich, Germany)	Reciprocating	2	1
HyFlex EDM (HF-EDM; Coltene/Whaledent AG, Altstätten, Switzerland)	Continuous	1	
TruNatomy (TrN; Dentsply-Maillefer, Ballaigues, Switzerland)	Continuous	1	1
Solite RS3 (S-RS3; Kedo Dental, Chennai, India)	Continuous	5	
Solite RS3 Black (S-RS3B; Kedo Dental, Chennai, India)	Continuous	1	
Solite RE Black (S-REB; Kedo Dental, Chennai, India)	Continuous	1	
Mtwo rotary (Mtwo; Seden & Martina, Padua, Italy)	Continuous		4
ProTaper Gold (PTG; Dentsply-Maillefer, Ballaigues, Switzerland)	Continuous		1
Hero Shaper (Micro-Mega, Besançon, France)	Continuous		1
IRaCe (FKG Dentaire, La Chaux-de-Fonds, Switzerland)	Continuous		1

## Data Availability

No new data were created or analyzed in this study. Data sharing is not applicable to this article.

## Abbreviations

The following abbreviations are used in this manuscript:

ISs	incisors
ICs	central incisors
ILs	lateral incisors
Ca	canines
PMs	premolars
Ms	molars
mx	maxillary
mb	mandibular
LAM	master apical file
GAM	master apical gutta-percha
US	ultrasonic tip
EA	apical extrusion
AIs	intraoperative accidents
LC	canal cleaning
DD	dentinal structural damage
T	time
It	instrumentation
O	obturation
GP	gutta-percha
ZOE	zinc oxide eugenol-based sealer
s	second
min	minute
OSM	stereomicroscope
SEM	scanning electron microscope
rx	radiograph
T1	time to reach working length
T2	time to completely remove obturation material
Tt	T1 + T2
PTUR	ProTaper Universal Retreatment files
H	Hedstrom file
K	K file
TFs	twisted files
TFA	twisted file adaptive files
PTU	ProTaper Universal files
PTN	ProTaper Next files
PTG	ProTaper Gold files
Re-Endo	Endostar RE Re-Endo files
GPR	NRT-GPR files
WO	Wave One files
WOG	Wave One Gold files
XP-ES	XP-endo Shaper files
XP-EFR	XP-endo Finisher files
NeoendoR	NeoEndo Retreatment files
EF-XRR	Edge-File XR Retreatment files
EF-X3	EdgeFile X3 files
HFR	HyFlex Remover files
RB	Reciproc Blue files
EF-EDM	HyFlex EDM files
TrN	TruNatomy files
S-RS3	Solite RS3 files
S-RS3B	Solite RS3 Black files
S-REB	Solite RE Black files

## Appendix A

**Table A1:** Risk of bias assessment of the included studies following the QUIN criteria.

Artículo	Item 1	Item 2	Item 3	Item 4	Item 5	Item 6	Item 7	Item 8	Item 9	Item 10	Item 11	Item 12	$\mathrm{Risk}\;\mathrm{of}\;\mathrm{Bias}\;(\%)=\frac{total\times 100}{\begin{array}{c} 2\times applicable\\ criteria \end{array}}$
Giuliani, Cocchetti y Pagavino (2008) [4]	2	0	0	**-**	1	1	0	2	1	1	1	2	50% moderate
Somma et al. (2008) [8]	2	0	2	-	1	0	0	1	1	1	1	2	50% moderate
Çelik Ünal et al. (2009) [12]	2	0	2	-	1	1	0	2	2	0	1	2	59.09% moderate
Marfisi et al. (2010) [13]	2	0	2	-	2	0	0	2	1	1	1	2	59.09% moderate
Uezu et al. (2010) [14]	2	0	0	-	2	0	0	2	0	0	1	2	40.91% high
Reddy et al. (2011) [15]	2	0	2	-	1	2	1	1	1	1	1	2	63.63% moderate
Shemesh et al. (2011) [16]	2	0	1	1	1	1	0	1	1	1	2	2	54.17% moderate
Siotia, Acharya y Gupta (2011) [17]	2	0	1	-	1	1	1	1	0	0	2	2	50% moderate
Yilmaz, Karapinar and Ozcelik (2011) [18]	2	0	0	-	1	1	1	2	-	0	1	2	50% moderate
Akhavan et al. (2012) [19]	2	0	0	-	1	0	1	2	-	0	1	2	45% high
Marques da Silva et al. (2012) [20]	2	0	2	-	1	1	1	2	0	0	1	2	54.55% moderate
Mollo et al. (2012) [21]	2	0	2	2	1	2	0	1	1	2	2	2	70.83% low
Solomonov et al. (2012) [22]	2	0	2	-	2	2	2	2	-	2	2	2	90% low
Voet et al. (2012) [23]	2	0	2	2	1	0	2	1	0	0	2	2	58.33% moderate
Reddy et al. (2013) [24]	2	0	2	-	2	0	0	2	0	0	1	2	50% moderate
Shivanand et al. (2013) [25]	2	0	2	-	2	0	1	2	0	0	1	2	54.55% moderate
Yadav et al. (2013) [26]	2	0	2	-	1	0	1	1	1	2	1	2	59.09% moderate
Chandrasekar et al. (2014) [2]	2	0	2	-	1	0	1	2	0	0	1	2	50% moderate
İriboz y Sazak Öveçoğlu (2014) [27]	2	0	2	-	2	1	1	2	1	0	2	1	63.64% moderate
Keles et al. (2014) [28]	2	0	2	-	1	1	2	2	-	0	2	2	70% moderate
Keleş et al. (2014) [29]	2	0	2	2	1	1	1	1	0	1	1	2	58.33% moderate
Topçuoǧlu et al. (2014) [30]	2	0	2	2	1	1	0	1	0	0	2	2	54.17% moderate
Topçuoğlu et al. (2014) [31]	2	0	2	2	1	1	1	1	1	2	2	2	70.83% low
Üstün et al. (2015) [32]	2	0	2	2	2	1	0	1	1	1	2	2	66.67% moderate
Çiçek et al. (2016) [33]	2	0	1	-	1	1	1	2	-	1	2	2	65% moderate
Joseph et al. (2016) [34]	2	0	1	-	1	1	1	2	1	1	1	2	59.09% moderate
Kanaparthy (2016) [35]	2	0	2	-	2	1	0	1	0	0	2	2	54.55% moderate
Kasam and Mariswamy (2016) [36]	2	0	2	2	1	0	1	2	0	0	2	2	58.33% moderate
Pawar et al. (2016) [37]	2	0	1	2	1	0	1	2	0	0	2	2	54.17% moderate
Preetam et al. (2016) [38]	2	1	1	-	1	0	0	2	0	0	2	2	50% moderate
Monardes et al. (2016) [39]	2	0	2	-	1	0	1	1	2	0	1	2	54.55% moderate
Das et al., (2017) [40]	2	0	2	-	1	1	1	2	0	0	1	2	54.55% moderate
Kayahan et al. (2017) [41]	2	1	1	-	1	1	1	1	-	0	1	2	55% moderate
Kesim et al. (2017) [42]	2	0	2	-	1	1	1	1	1	2	2	2	68.18% moderate
Özyürek et al. (2017) [43]	2	0	2	2	2	1	1	2	2	2	2	2	83.33% low
Jain, Nikhil and Bansal (2018) [44]	1	0	2	-	1	0	1	1	0	0	2	2	45.45% high
Jena et al. (2018) [45]	2	2	2	-	2	0	1	2	-	0	1	2	70% moderate
Kakoura and Pantelidou (2018) [46]	2	0	2	2	2	1	2	2	1	2	2	2	83.33% low
Raj et al. (2018) [47]	2	0	2	-	1	1	1	2	-	0	2	2	65% moderate
Rödig et al. (2018) [48]	2	0	2	-	1	1	0	2	1	0	1	2	54.55% moderate
Agrawal et al. (2019) [49]	2	0	2	-	1	0	1	2	-	0	2	2	60% moderate
Ali et al. (2019) [50]	2	2	2	-	2	1	1	2	-	0	2	2	80% low
Kulkarni et al. (2019) [51]	2	0	0	-	1	1	1	1	0	0	2	2	45.45% high
Hasija et al. (2020) [52]	2	0	2	2	1	0	1	1	1	2	1	2	62.5% moderate
Maruster et al. (2020) [53]	2	0	-	-	1	0	1	1	0	0	1	2	40% high
Mutar y Al-Zaka (2020) [54]	2	0	1	-	1	0	0	2	-	0	2	2	50% moderate
Purba et al. (2020) [55]	2	0	2	-	2	1	1	2	1	2	2	2	77.27% low
Topçuoğlu et al. (2020) [56]	2	2	1	2	2	1	1	2	-	0	2	2	77.27% low
AbuMostafa, Almoqayyad y Mohammad (2021) [57]	2	2	2	2	2	1	1	2	0	0	2	2	75% low
Ali et al. (2021) [58]	1	2	2	-	2	2	1	2	0	0	2	2	72.73% low
Colmenar et al. (2021) [59]	2	0	1	-	1	1	1	1	-	0	1	2	50% moderate
Eid, Maksoud and Elsewify (2021) [60]	2	0	2	-	1	1	1	1	0	0	2	2	54.55% moderate
Muraleedhar et al. (2021) [61]	2	0	1	-	1	0	1	1	-	0	2	2	50% moderate
Özlek and Gündüz (2021) [62]	1	1	2	-	1	0	1	1	0	0	2	2	50% moderate
Subramanian et al. (2021) [63]	2	0	2	-	1	0	1	1	0	0	2	2	50% moderate
Das et al. (2022) [64]	2	0	2	1	2	0	1	2	0	0	2	2	58.33% moderate
Hyun et al. (2022) [65]	2	0	2	-	2	2	1	1	-	0	2	2	70% moderate
Sameh and Omaia (2022) [3]	2	0	2	-	1	0	2	1	1	1	2	2	63.63% moderate
Shaheen, Elhelbawy and Sherif (2022) [6]	2	0	2	2	1	1	1	1	0	0	2	2	58.33% moderate
Tejaswi et al. (2022) [66]	2	0	2	2	1	0	0	1	0	0	2	2	50% moderate
Abdelnaby, Ibrahim and ElBackly (2023) [67]	2	2	2	-	2	1	2	2	2	2	2	2	95.45% low
Akshay et al. (2023) [5]	2	0	2	-	1	0	1	1	0	0	2	1	45.45% high
Baig et al. (2023) [1]	2	0	2	-	1	0	1	2	0	0	2	2	54.55% moderate
Kim, Chang and Oh (2023) [9]	2	1	-	-	1	1	0	1	0	0	2	2	50% moderate
Rama Sowmya et al. (2023) [68]	2	0	2	-	1	1	2	1	-	0	2	2	65% moderate
Tsenova-Ilieva et al. (2023) [69]	2	0	2	-	1	2	-	2	1	2	2	2	80% low
Varghese et al. (2023) [7]	2	0	2	-	1	0	1	0	0	0	2	2	45.45% high
Aarthi et al. (2024) [70]	2	0	2	2	1	0	0	2	0	0	2	1	50% moderate
Abboud et al. (2024) [71]	2	2	2	-	2	1	2	2	-	0	2	2	85% low
Buyuksungur et al. (2024) [72]	2	2	2	-	1	1	1	2	-	0	2	2	75% low
Karunakar et al. (2024) [73]	2	0	2	-	1	1	1	2	0	0	1	1	50% moderate
Nour, Elgendy and Bayoumi (2024) [74]	2	2	2	-	2	0	0	2	-	0	2	2	70% moderate
Sairaman et al. (2024) [75]	2	0	2	-	2	1	1	1	-	0	2	2	65% moderate
Sairaman et al. (2024) [76]	2	1	2	-	2	1	1	2	-	0	2	2	75% low
Sankar et al. (2024) [77]	2	0	2	-	1	1	2	1	1	0	2	2	63.63% moderate
Singh et al. (2024) [78]	2	0	2	-	1	0	1	1	0	0	2	1	45.45% high
Suresh et al. (2024) [79]	2	2	2	-	1	1	2	2	2	0	2	2	81.82% low
Yadav et al. (2024) [80]	2	0	2	-	1	0	1	1	0	0	1	2	45.45% high
	1	2	3	4	5	6	7	8	9	10	11	12	

Note: 1—objectives; 2—sample size calculation; 3—sampling technique; 4—details of the comparison/control group; 5—explanation of the methodology; 6—operator details; 7—randomization; 8—outcome measures; 9—outcome assessor details; 10—blinding; 11—statistical analysis; 12—results presentation. Response options: ‘-’, not applicable; 0, not specified; 1, inadequately specified; 2, adequately specified.
